# Reviewing the connection between speech and obstructive sleep apnea

**DOI:** 10.1186/s12938-016-0138-5

**Published:** 2016-02-20

**Authors:** Fernando Espinoza-Cuadros, Rubén Fernández-Pozo, Doroteo T. Toledano, José D. Alcázar-Ramírez, Eduardo López-Gonzalo, Luis A. Hernández-Gómez

**Affiliations:** GAPS Signal Processing Applications Group, Universidad Politécnica de Madrid, Madrid, Spain; ATVS Biometric Recognition Group, Universidad Autónoma de Madrid, Madrid, Spain; Respiratory Department, Sleep Unit Hospital Quirón Málaga, Málaga, Spain

**Keywords:** Obstructive sleep apnea, Speech, Clinical variables, Speaker’s voice characterization, Supervector, Gaussian mixture models, i-vector, Support vector regression

## Abstract

**Background:**

Sleep apnea (OSA) is a common sleep disorder characterized by recurring breathing pauses during sleep caused by a blockage of the upper airway (UA). The altered UA structure or function in OSA speakers has led to hypothesize the automatic analysis of speech for OSA assessment. In this paper we critically review several approaches using speech analysis and machine learning techniques for OSA detection, and discuss the limitations that can arise when using machine learning techniques for diagnostic applications.

**Methods:**

A large speech database including 426 male Spanish speakers suspected to suffer OSA and derived to a sleep disorders unit was used to study the clinical validity of several proposals using machine learning techniques to predict the apnea–hypopnea index (AHI) or classify individuals according to their OSA severity. AHI describes the severity of patients’ condition. We first evaluate AHI prediction using state-of-the-art speaker recognition technologies: speech spectral information is modelled using supervectors or i-vectors techniques, and AHI is predicted through support vector regression (SVR). Using the same database we then critically review several OSA classification approaches previously proposed. The influence and possible interference of other clinical variables or characteristics available for our OSA population: age, height, weight, body mass index, and cervical perimeter, are also studied.

**Results:**

The poor results obtained when estimating AHI using supervectors or i-vectors followed by SVR contrast with the positive results reported by previous research. This fact prompted us to a careful review of these approaches, also testing some reported results over our database. Several methodological limitations and deficiencies were detected that may have led to overoptimistic results.

**Conclusion:**

The methodological deficiencies observed after critically reviewing previous research can be relevant examples of potential pitfalls when using machine learning techniques for diagnostic applications. We have found two common limitations that can explain the likelihood of false discovery in previous research: (1) the use of prediction models derived from sources, such as speech, which are also correlated with other patient characteristics (age, height, sex,…) that act as confounding factors; and (2) overfitting of feature selection and validation methods when working with a high number of variables compared to the number of cases. We hope this study could not only be a useful example of relevant issues when using machine learning for medical diagnosis, but it will also help in guiding further research on the connection between speech and OSA.

## Background

Sleep disorders are receiving increased attention as a cause of daytime sleepiness, impaired work, traffic accidents, and are associated with hypertension, heart failure, arrhythmia, and diabetes. Among sleep disorders, obstructive sleep apnea (OSA) is the most frequent one [1]. OSA is characterized by recurring episodes of breathing pauses during sleep, greater than 10 s at a time, caused by a blockage of the upper airway (UA) at the level of the pharynx.

The gold standard for sleep apnea diagnosis is the polysomnography (PSG) test [2]. This test requires an overnight stay of the patient at the sleep unit within a hospital to monitor breathing patterns, heart rhythm and limb movements. As a result of this test, the apnea–hypopnea index (AHI) is computed as the average number of apnea and hypopnea episodes (partial and total breath cessation episodes respectively) per hour of sleep. Because of its high reliability this index is used to describe the severity of patients’ condition: low AHI (AHI <10) indicates a healthy subject or mild OSA patient (10≤ AHI ≤30), while AHI above 30 is associated with severe OSA. Waiting lists for PSG may exceed 1 year in some countries as Spain [3]. Therefore, faster and less costly alternatives have been proposed for early OSA detection and severity assessment; and speech-based methods are among them.

The rationale of using speech analysis in OSA assessment can be found on early works such as the one by Davidson et al. [4] where the evolutionary changes in the acquisition of speech are connected to the appearance of OSA from an anatomical basis. Several studies have shown physical alterations in OSA patients such as craniofacial abnormalities, dental occlusion, longer distance between the hyoid bone and the mandibular plane, relaxed pharyngeal soft tissues, large tongue base, etc. that generally cause a longer and more collapsible upper airway (UA). Consequently, abnormal or particular speech features in OSA speakers may be expected from an altered structure or function of their UA.

Early approaches to speech-based OSA detection can be found in [5] and [6]. In [5] authors used perceptive speech descriptors (related to articulation, phonation and resonance) to correctly identify 96.3 % of normal (healthy) subjects, though only 63.0 % of sleep apnea speakers were detected. The use of acoustic analysis of speech for OSA detection was first presented in [7] and [8]. Fiz et al. [7] examined the harmonic structure of vowels spectra, finding a narrower frequency range for OSA speakers, which may point at differences in laryngeal behavior between OSA and non-OSA speakers. Later on, Robb et al. [8] presented an acoustic analysis of vocal tract formant frequencies and bandwidths, thus focusing on the supra-laryngeal level where OSA-related alterations should have larger impact according to the pathogenesis of the disorder.

These early contributions have driven recent proposals for using automatic speech processing in OSA detection such as [9–14]. Different approaches, generally using machine learning techniques, have been studied for Hebrew [9, 14] and Spanish [10–13] languages. Results have been reported for different types of speech (i.e., sustained and/or continuous speech) [9, 11, 13], different speech features [9, 12, 13], and modeling different linguistic units [11]. Also speech recorded from two distinct positions, upright or seated and supine or stretched, has been considered [13, 15].

Despite the positive results reported in these previous studies (including ours), as it will be presented in the section “Discussion”, we have found contradictory results when applying the proposed methods over our large clinical database composed of speech samples from 426 OSA male speakers. The next section describes a new method for estimating the AHI using state-of-the-art speaker’s voice characterization technologies. This same approach has been recently tested and demonstrated to be effective in the estimation of other characteristics in speakers’ populations such as age [16] and height [17]. However, as it can be seen in the section “Results”, only a very limited performance is found when this approach is used for AHI prediction. These poor results contrast with the positive results reported by previous research and motivated us to their careful review. The review (presented in the section “Discussion”) reveals some common limitation and deficiencies when developing and validating machine learning techniques, as overfitting and false discovery (i.e., finding spurious or indirect associations) [18], that may have led to overoptimistic previous results. Therefore, our study can represent an important and useful example to illustrate the potential pitfalls in the development of machine learning techniques for diagnostic applications as it is being identified by the biomedical engineering research community [19].

As we conclude at the end of the paper, we not only hope that our study could be useful to help in the development of machine learning techniques in biomedical engineering research, we also think it can help in guiding any future research on the connection between speech and OSA.

## Methods

### Subjects and experimental design

The population under study is composed by 426 male subjects presenting symptoms of OSA during a preliminary interview with a pneumonologist: such as excessive daytime sleepiness, snoring, choking during sleep, or somnolent driving. Several clinical variables were collected for each individual: age, height, weight, body-mass index (BMI, defined as the weight in kilograms divided by the square of the height in meters, kg/m^2^) and cervical perimeter (CP, measure of the neck circumference, in centimeters, at the level of the cricothyroid membrane). This database has been recorded at the Hospital Quirón de Málaga (Spain) since 2010 and is, to the best of our knowledge, the largest database used in this kind of studies. The database contains 597 speakers: 426 males and 171 females. Our study had no impact on the diagnosis process of patients or on their possible medical treatment therefore the Hospital did not consider necessary to seek approval from their ethics committee. Before starting the study, participants were notified about the research and their informed consent obtained. Statistics of the clinical variables for the male population in this study are summarized in Table 1.

**Table 1:** Descriptive statistics on the 426 male subjects

Clinical variables	Mean	SD	Range
AHI	22.5	18.1	0.0–102.0
Weight (kg)	91.7	17.3	61.0–162.0
Height (cm)	175.3	7.1	152.0–197.0
BMI (kg/m^2^)	29.8	5.1	20.1–52.1
Age (years)	48.8	12.5	20.0–85.0
Cervical perimeter (cm)	42.2	3.2	34.0–53.0

*AHI* apnea–hypopnea index, *BMI* body mass index, *SD* standard deviation

The diagnosis for each patient was confirmed by specialized medical staff through PSG, obtaining the AHI on the basis of the number of apnea and hypopnea episodes. Patients’ speech was recorded prior to PSG. All speakers read the same 4 sentences and sustained a complete set of Spanish vowels [i.e., a, o, u]. Sentences were designed trying to cover relevant linguistic/phonetic contexts related to peculiarities in OSA voices (see details in [12]). Recordings were made in a room with low noise and patients at an upright or seated position. Recording equipment was a standard laptop with an USB SP500 Plantronics headset. Speech was recorded at a sampling frequency of 50 kHz and encoded in 16 bits. Afterwards it was down-sampled to 16 kHz before processing.

### Problem formulation

Our major aim is testing whether state-of-the-art speaker’s voice characterization technologies that have already demonstrated to be effective in the estimation of speaker’s characteristics such as age [16] and height [17] could be also effective in estimating the AHI. It is important to point out that, besides predicting the AHI from speech samples, we also tested the performance when using these same techniques to estimate the other clinical variables (age, height, weight, BMI and CP). We think this evaluation is relevant for two main reasons: firstly, to validate our methodology, by comparing our results estimating age, height and BMI with those previously reported over general speaker populations (such as [16, 17, 20]); and secondly, to identify correlations between speech and other clinical variables that can increase the likelihood of false discovery based on spurious or indirect associations [18] between these clinical variables and AHI. This second aspect we will be relevant when presenting the critical review of previous approaches to OSA assessment in the section “Discussion”.

Consequently, our study can be formulated as a machine learning regression problem as follows: we are given a training dataset of speech recordings and clinical variables information ${\mathbf{S}}_{\text{tr}}^{j} = \left\{ {{\mathbf{x}}_{n} , y_{n}^{j} } \right\}_{n = 1}^{N}$, where ${\mathbf{x}}_{n} \in \Re^{\text{p}}$ denotes the acoustic representation for the nth utterance of the training dataset and $y_{n}^{j} \in \Re$ denotes the corresponding value of the clinical variable for the speaker of that utterance; *j* corresponds to a particular variable in the set of V clinical variables (*j* = 1, 2, …V; i.e., AHI, age, height, weight, BMI, CP).

The goal is to design an estimator function $f^{j}$ for each clinical variable, such that for an utterance of an unseen testing speaker **x**_tst_, the difference between the estimated value of that particular clinical variable $\hat{y}^{j} = f^{j} \left( {{\mathbf{x}}_{{\text{tst}}} } \right)$ and its actual value $y^{j}$ is minimized.

Once this regression problem has been formulated two main issues must be addressed: 1) what acoustic representation and model will be used for a given utterance **x**_*n*_ and 2) how to design the regression or estimator functions $f^{j}$.

### Acoustic representation of OSA-related sounds

Besides the linguistic message, speech signals carry important information about speakers mainly related to their particular physical or physiological characteristics. This has been the basis for the development of automatic speaker recognition systems, automatic detection of vocal fold pathologies, emotional/psychological state recognition as well as age and weight (or BMI) estimation. In a similar vein, the specific characteristics of the UA in OSA individuals have led to hypothesize OSA detection using automatic acoustic analysis of speech sounds.

To represent OSA-specific acoustic information, speech records in our database include read speech of four sentences that were designed to contain specific distinctive sounds to discriminate between healthy and OSA speakers. The design of these four sentences was done according to the reference research in [5] and [6], where Fox et al. identify a set of speech descriptors in OSA speakers related to articulation, phonation and resonance. For example, the third sentence in our corpus includes mostly nasal sounds to detect the expected resonance anomalies in OSA individuals (the details on the design criteria for this corpus can be found in [12]). Additionally, to exclude any acoustic factor not related to OSA discrimination, the speech signal acquisition was done in a room with low noise and using a single high quality microphone (USB SP500 Plantronics headset).

**Fig. 1:**
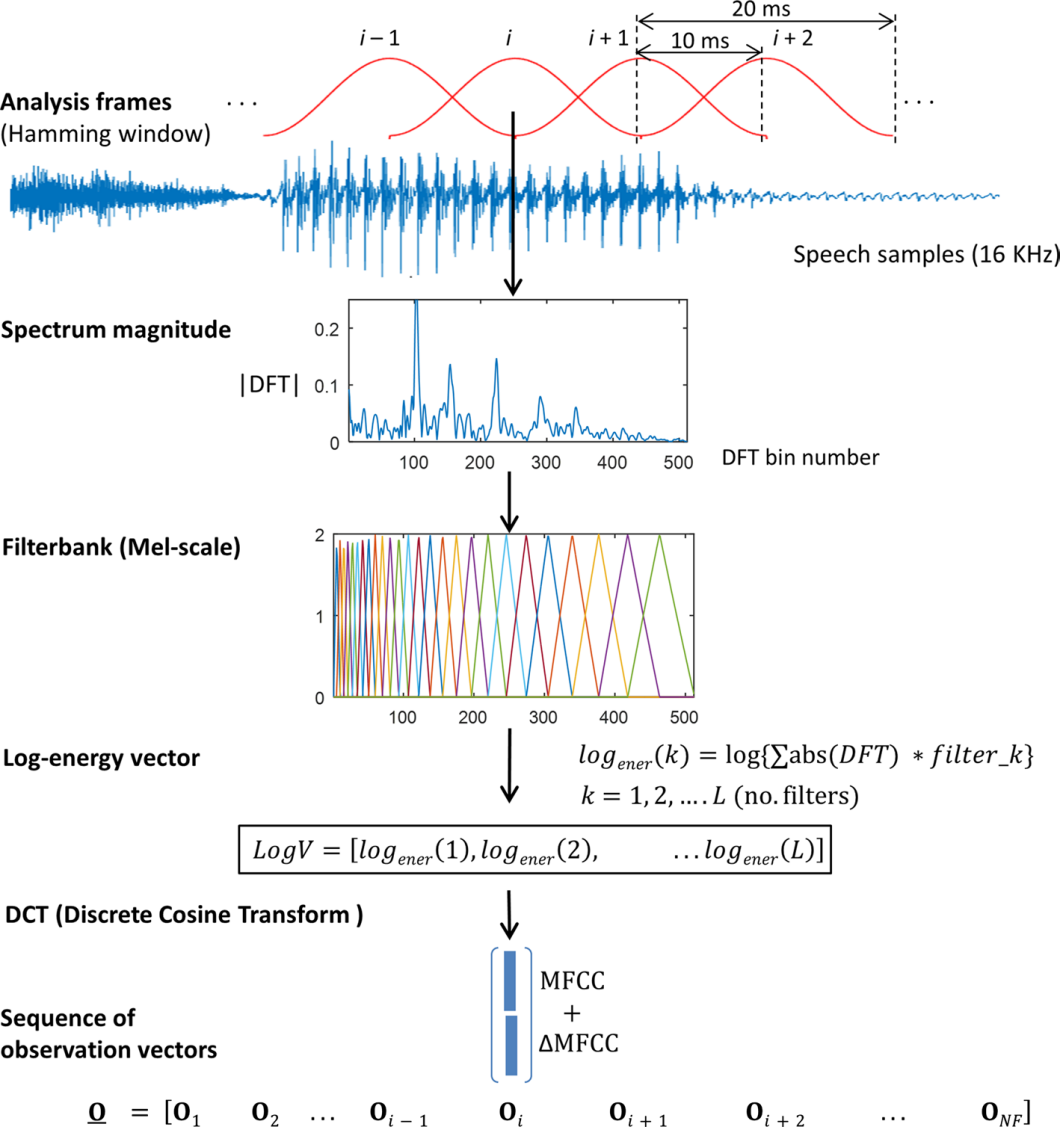
Acoustic representation of utterances

Once we have a set of speech utterances containing OSA-specific sounds and collected under a controlled recording environment, speech signals were processed at a sampling frequency of 16 kHz to have a precise wide-band representation all the relevant information in the speech spectrum. As Fig. 1 illustrates, each sentence was analyzed in speech segments (i.e., frames) of 20 ms duration with an overlap of 10 ms; each speech frame was multiplied by a Hamming window. The spectral envelope of each frame was then represented using mel-frequency cepstral coefficients (MFCCs). MFCCs provide a spectral envelope representation of speech sounds extensively used in automatic speech and speaker recognition [21, 22], pathological voice detection, age, height and BMI estimation [16, 17, 20], etc. MFCCS have also been used in previous research on speech-based OSA detection [9–11] and [14].

In the MFCC representation the spectrum magnitude of each speech frame is first obtained as the absolute value of its DFT (discrete Fourier transform). Then a filterbank of triangular filters spaced in a frequency scale based on the human perception system (i.e., Mel-scale) is used to obtain a vector with the log-energies of each filter (see Fig. 1). Finally, a discrete cosine transform (DCT) is applied over the vector of log filterbank energies to produce a compact set of decorrelated MFCC coefficients. Additionally, in order to represent the spectral change over time, MFFCs are extended to their first order (velocity or delta ΔMFCCs) time derivatives (more details on MFCCs parametrization can be found in [23]). So far, in our experiments, in each speech frame *i* the acoustic information is represented by a D-dimensional vector **O**_*i*_, called observation vector, that includes 19 MFFCs +19 ΔMFCCs parameters, thus D = 38. The extraction of MFCCswas performed using the HTK software (htk.eng.cam.ac.uk), see Table 2 for the details on DFT order, number of triangular filters, etc.

**Table 2:** Implementation tools

Tool^a^	Function name	Function description	Parameters
HTK	HCopy	Extract the MFCCs coefficients	No. DFT bins = 512
			No. filters = 26
			No. MFCC coeff. = 19
			No. ΔMFCC coeff. = 19
MSR Identity ToolBox^b^	GMM_em	GMM–UBM training	No. mixtures = 512
			No. of expectation maximization iteration = 10
			Feature sub-sampling factor = 1
	MapAdapt	GMM adaptation	Adaptation algorithm = MAP
			No. mixtures = 512
			MAP relevance factor = 10
	Train_tv_space	Total variability matrix training	Dimension of total variability matrix = {400,300,200,100,50,30}
			Number of iteration = 5
	Extract_ivector	I-vector training	Dimension of total variability matrix = {400,300,200,100,50,30}
LIBSVM	SVM_train	SVR training	Grid search parameters: C, model complexity = −20:20 $\in$, insensitive-zone = 2^−7^:2^7^
	SVM_predict	SVR regression	Grid search parameters: C, model complexity = −20:20 $\in$, insensitive-zone = 2^−7^:2^7^

^a^All the implementation tools were used under Linux Ubuntu 12.04 LTS Operating System

^b^Executed on Matlab 2014a

### Utterance modelling

Due to the natural variability in speech production different utterances corresponding to the same sentence will exhibit variable-duration and thus will be represented by a variable-length sequence **O** of observation vectors:

1 $$\underline{{\mathbf{O}}} = \left[ {{\mathbf{O}}_{1} ,\;{\mathbf{O}}_{2} \ldots {\mathbf{O}}_{NF} } \right]$$

where **O**_*i*_ is the D-dimensional observation vector at frame *i* and *NF* is the number of frames, which will be variable due to the different durations when reading the same sentence. This variable-length sequence cannot be the input for a regression algorithm as support vector regression (SVR) that will be the estimator function $f^{j}$ to predict $y^{j}$ (being $y^{j}$ the AHI and the other clinical variables: age, height, weight, BMI and CP).

Consequently, the sequence of observations **O** must be mapped into a vector with fixed dimension. In our method, this has been done using two modeling approaches, referred to as supervectors and i-vectors, which have been successfully applied to speaker recognition [24], language recognition [25], speaker age estimation [16], speaker height estimation [17] and accent recognition [26]. We think that their success in those challenging tasks were speech contains significant sources of interfering intra-speaker variability (speaker weight, height, etc.), is a reasonable guarantee for exploring its use in estimating the AHI and other clinical variables in our OSA population.

It is also important to point out that we have avoided the use of feature selection procedures because, as it will be commented in the section “Discussion”, we believe this has led to over-fitted results in several previous studies in this field. It is for that reason that in our approach we evaluate high-dimensional acoustic modelling provided by supervectors and low-dimensional i-vectors representations based on subspace projection. These two techniques are described below.

### Supervectors

**Fig. 2:**
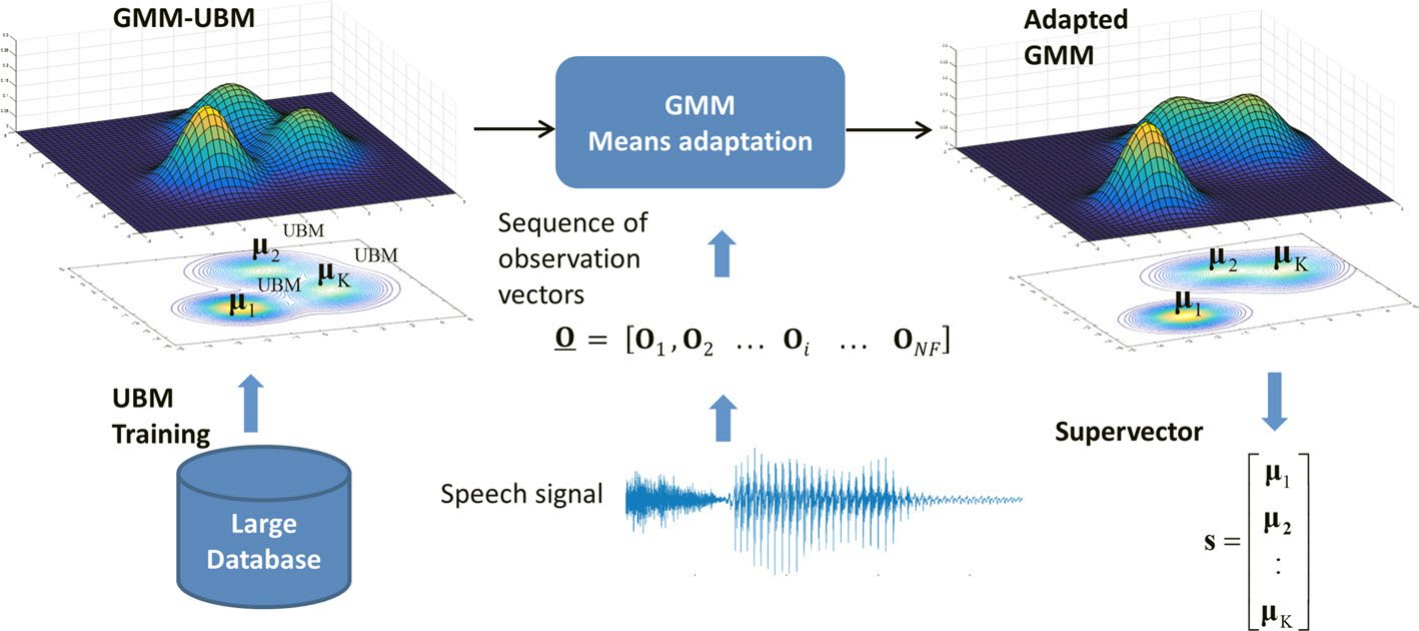
GMM and supervector modelling

Both supervector and i-vector modelling approaches start by fitting a Gaussian mixture model (GMM) to the sequence of observations **O**. A GMM (see [23, 27]) consists of a weighted sum of K D-dimensional Gaussian components, where, in our case, D is the dimension of the MFFCs observation vectors. Each *i*-th Gaussian component is represented by a mean vector (**µ**_*i*_) of dimension D and a D × D covariance matrix (**Σ**_*i*_). Due to limited data, it is not possible to accurately fit a separate GMM for a short utterance, specially when using a high number of Gaussian components (i.e., large K). Consequently, GMMs are obtained using adaptation techniques from a universal background model (UBM), which is also a GMM trained on a large database containing speech from a large number of different speakers [23]. Therefore, as Fig. 2 illustrates, the variable-length sequence **O** of vectors of a given utterance is used to adapt a GMM–UBM generating an adapted GMM where only the means (**µ**_*i*_) are adapted.

In the supervector modelling approach [21], the adapted GMM means (**µ**_*i*_) are extracted and concatenated (appending one after the other) into a single high-dimensional vector **s** that is called the GMM mean supervector:

2 $${\mathbf{s}} = \left[ {\begin{array}{*{20}c} {{\mathbf{\mu }}_{{\text{1}}} } \\ {{\mathbf{\mu }}_{{\text{2}}} } \\ \vdots \\ {{\mathbf{\mu }}_{K} } \\ \end{array} } \right]$$

The resulting fixed-length supervector, of size K × D, is now suitable to be used as input to a regression algorithm, such as SVR, to predict AHI and the other clinical variables.

As it is summarized in Table 2, in our experiments GMM–UBM training, GMM adaptation and supervector generation was done using the MSR Identity ToolBox for Matlab™ [28] running over Matlab 2014a on Linux Ubuntu 12.04 LTS. As it is also shown in Table 2, to have a precise acoustic representation for each sentence a GMM with K = 512 components was used, resulting in a high-dimensional supervector of size K × D = 19,456 = 38 × 512 (D = 38 is the dimension of MFFCs observation vectors **O**_*i*_).

As mentioned before, training the GMM UBM requires a considerable amount of development data to represent a global acoustic space. Therefore, for development we used several large databases containing microphonic speech sampled at 16 kHz, covering a wide range of phonetic variability from continuous/read Spanish speech (see, for example, ALBAYZIN [29], as it was one the databases we used). The whole development dataset includes 25,451 speech recordings from 940 speakers. Among them 126 speakers certainly diagnosed with OSA, and not used for tests, were also included to reflect OSA-specific characteristics of speech.

### I-vectors

Beyond the success of high-dimensional supervectors, a new paradigm called i-vector has been successfully and is widely used by the speaker recognition community [24]. The i-vector model relies on the definition of a low-dimensional total variability subspace and can be described in the GMM mean supervector space by:

3 $${\mathbf{s}} = {\mathbf{m}} + {\mathbf{Tw}}$$

where **s** is the GMM mean supervector representing an utterance and **m** is the mean supervector obtained from the UBM GMM–UBM, which can be considered a global acoustic representation independent from utterance, speaker, health and clinical condition. **T** is a rectangular low rank matrix representing the primary directions of total acoustic variability observed in a large development speech database, and **w** is a low dimensional random vector having a standard normal distribution. In short, Eq. (3) can be viewed as a simple factor analysis for projecting the high-dimensional (in order of thousands) supervector **s** to the low-dimensional (in order of hundreds) factor vector, identity vector or i-vector **w**. **T** is named the total variability matrix and the components of i-vector **w** are the total factors that represent the acoustic information in the reduced total variability space. Compared to supervectors, the total variability modeling using i-vectors has the advantage of projecting the high dimensionality of GMM supervectors into a low-dimensional subspace, where most of the speaker-specific variability is captured.

Automatic speech recognition systems typically use i-vectors with dimensionality of 400. In our tests the total variability matrix **T** was estimated using the same development data described before for training the GMM–UBM, and we evaluated subspace projections for i-vectors with different dimensions ranging from 30 to 400. Efficient procedure for training **T** and MAP adaptation of i-vectors can be found in [30]. In our tests we use the implementation provided by MSR Identity ToolBox for Matlab™ [28] running over Matlab 2014a on Ubunutu 12.04 LTS (see the details in Table 2).

### Regression using SVR

Once an utterance is represented by a fixed-length vector, supervector or i-vector, SVR is employed as the estimator function $f^{j}$ to predict $y^{j}$, i.e., the AHI and other clinical variables (age, height, weight, BMI and CP).

SVR is a function approximation approach developed as a regression version of the widely known Support Vector Machine (SVM) classifier [31]. When using SVR, the input variable (i-vector/supervector) is firstly mapped onto a high dimensional feature space by using a non-linear mapping. The mapping is performed by the kernel function. The kernel yields the new high dimensional feature by a similarity measure between the points from the original feature space. Once the mapping onto a high dimensional space is done then a linear model is constructed in this feature space by finding the optimal hyperplane in which most of the of the training samples lie within an $\in$-margin ($\in$-insensitive zone) around this hyperplane [31].

The generalization of SVR’s performance depends on a good setting of two hyperparameters ($\in$, C) and the kernel parameters. The parameter $\in$ controls the width of the $\in$-insensitive zone, used to fit the training data. The width of the $\in$-insensitive zone determines the level of accuracy of approximation function. It relies entirely on the target values of the training set. The parameter C determines the trade-off between the model complexity, controlled by $\in$, and the degree to which deviations larger than the $\in$-insensitive zone are tolerated in the optimization of the hyperplane. Finally, the kernel parameters depend on the type similarity measure used.

In this paper, SVR is applied to estimate the clinical variables and linear and radial basis function (RBF) kernels were tested to approximate the estimator function $f^{j}$. In our study, both linear and RBF kernels were tested for i-vectors, but only linear kernels were considered for supervectors because their large dimensionality makes it not advisable mapping them into a higher dimensional space. SVR training and testing were implemented by using LIBSVM [32] running on Linux Ubuntu 12.04 LTS. Table 2 describes de details of use for this software together with all the parameters used in our tests.

### Performance metrics

To evaluate the proposed method of using supervectors or i-vectors to predict or estimate AHI and the other clinical variables (age, height, weight, BMI and CP) we measure both the mean absolute error (MAE) and the Pearson correlation coefficient (ρ). MAE provides the average absolute difference between actual and estimated values, while ρ evaluates their linear relationship. As we will see in the section “Results”, correlation coefficients between estimated and actual AHI values were many times very small. Therefore, we considered informative to report p-values for correlation coefficients as the probability that they were in fact zero (null hypothesis).

Although the main objective of our method is to evaluate the capability of using speech to predict or estimate AHI, in the section “Discussion” we also review previous research that aim at classify or discriminate between subjects with OSA (AHI ≥10) and without OSA (defined by an AHI <10). Therefore, we performed some additional tests using our estimated AHI values to classify subjects as OSA (predicted AHI ≥10) and non-OSA (predicted AHI <10). In these classification tests performance was measured in terms of sensitivity, specificity and the area under the ROC curve.

### *k*-fold cross-validation and grid-search

**Fig. 3:**
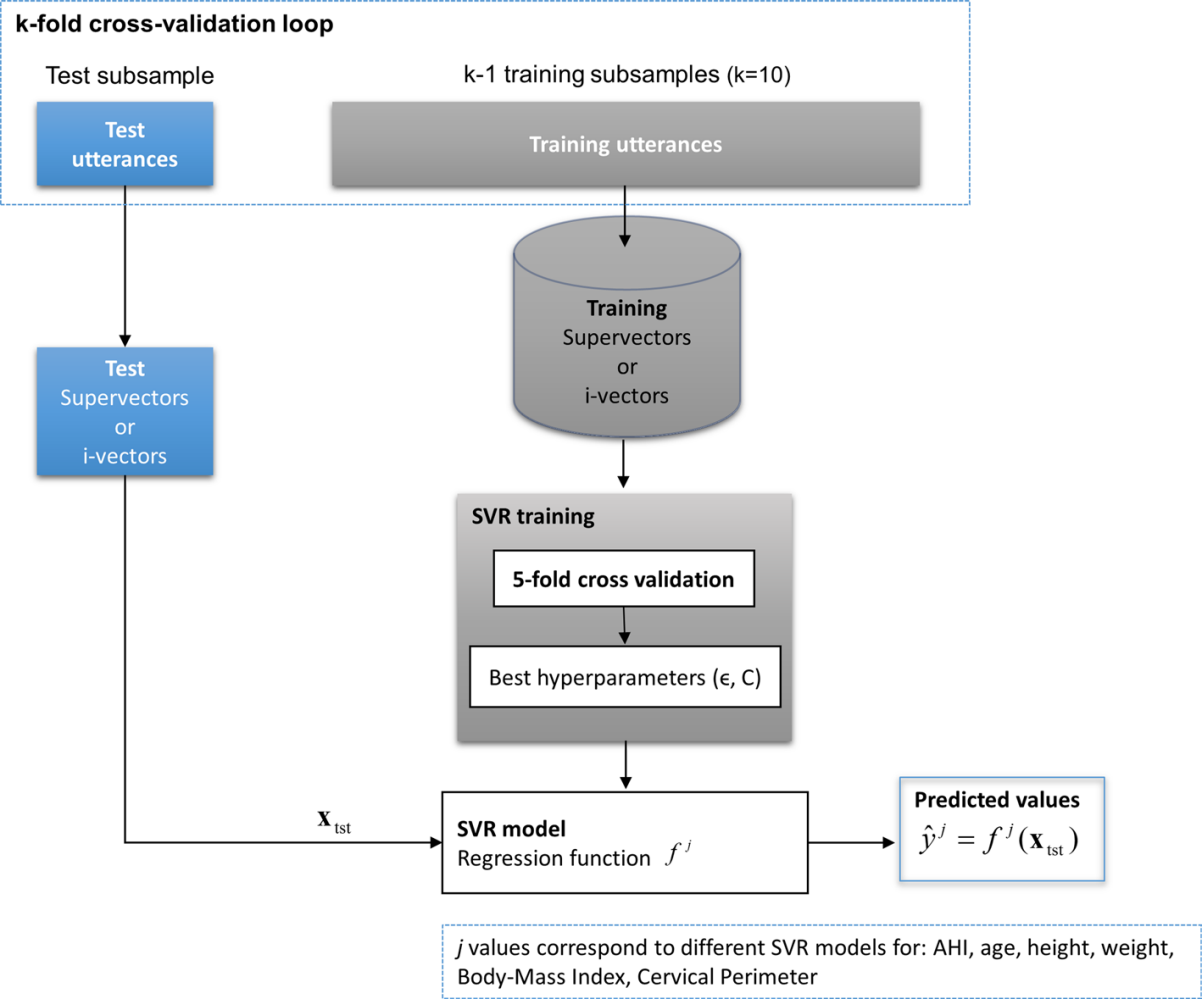
Representation of k-fold cross-validation and grid search for SVR regression and predicting clinical variables

In order to train the SVR regression model (function $f^{j}$) and predict $y^{j}$ variables (AHI and other clinical variables) we have employed k-fold cross-validation and grid-search for finding the optimal SVR parameters. The whole process is presented in Fig. 3. Firstly, to guarantee that all speakers are involved on the test, the dataset is split into k equal sized subsamples with no speakers in common. Then, of the k subsamples, a single subsample is retained for testing and the remaining k−1 subsamples are used as training dataset. Results were reported for k = 10.

Furthermore, as Fig. 3 also illustrates, in each cross-validation loop the optimal hyperparameters (ϵ, C) of the SVR models are obtained through “grid search” using a fivefold cross-validation on the training data. The ranges for this grid search are detailed in Table 2.

## Results

### Clinical variables estimation

Results in Tables 3 and 4 show performance when using speech to estimate age and height. As mentioned before, the purpose of these tests is to validate our procedure by comparing these results to those reported in recent references [16] and [17]. Table 3 shows that our estimation performance (both in terms of MAE and correlation coefficient) for height are comparable, and better when using i-vectors, that those in [17]. However estimation results for age, Table 4, are slightly worse than [16]. A plausible explanation is that the population in [16] includes a majority of young people, between 20 and 30 years old, while most of our OSA speakers are well above 45 years old. According to [16] speech records from young speakers can be better discriminated than those from older ones. In any case, our results are very similar to results published previously by other authors, which is a good indicator of the validity of our methods.

**Table 3:** Speakers’ height estimation results

Regression method	Mean absolute error (cm)	Correlation coefficient (ρ)
I-vector–LSSVR [17]	6.2	0.41^b^
Supervector–SVR	5.37	0.34^a^
I-vector–SVR	5.06	0.45^a^

^a^These values are significant beyond the 0.01 level of confidence

^b^Level of confidence is not reported

**Table 4:** Speakers’ age estimation results

Regression method	Mean absolute error (years)	Correlation coefficient (ρ)
I-vector–WCCN–SVR [16]	6.0	0.77^b^
Supervector–SVR	7.75	0.66^a^
I-vector–SVR	7.87	0.63^a^

^a^These values are significant beyond the 0.01 level of confidence

^b^Level of confidence is not reported

Prediction results using i-vectors and supervectors for all our clinical variables are listed on Tables 5, 6 and 7.

**Table 5:** Speakers’ clinical variables estimation using supervector-SVR (linear kernel)

Clinical variable	MAE	ρ
AHI	14.26	0.17
Height (cm)	5.37	0.34
Age (years)	7.75	0.66
Weight (kg)	12.58	0.31
BMI (kg/m^2^)	3.81	0.23
CP (cm)	2.29	0.42

*AHI* apnea–hypopnea index, *BMI* body mass index, *CP* cervical perimeter

The correlation coefficients (ρ) are significant beyond the 0.01 level of confidence

**Table 6:** Speakers’ clinical variables estimation using i-vectors-SVR (linear kernel)

Clinical variable	I-vector dimension											
	Mean absolute error (MAE)						Correlation coefficient (ρ)					
	400	300	200	100	50	30	400	300	200	100	50	30
AHI	13.68	13.64	13.55	13.23	13.40	13.85	0.23	0.21	0.24	0.30	0.27	0.20
Height (cm)	5.21	5.23	5.11	5.06	5.29	5.38	0.40	0.41	0.43	0.45	0.36	0.34
Age (years)	8.16	7.87	8.11	8.29	8.77	9.16	0.61	0.63	0.61	0.59	0.52	0.44
Weight (kg)	12.31	12.23	12.25	11.86	12.16	12.31	0.34	0.35	0.36	0.39	0.35	0.31
BMI (kg/m^2^)	3.59	3.65	3.67	3.69	3.74	3.80	0.33	0.30	0.29	0.28	0.26	0.18
CP (cm)	2.28	2.26	2.20	2.26	2.31	2.42	0.44	0.45	0.49	0.47	0.44	0.32

*AHI* apnea–hypopnea index, *BMI* body mass index, *CP* cervical perimeter

The correlation coefficients (ρ) are significant beyond the 0.01 level of confidence

**Table 7:** Speakers clinical variables estimation using i-vectors-SVR (RBF kernel)

Clinical variable	I-vector dimension											
	Mean absolute error (MAE)						Correlation coefficient (ρ)					
	400	300	200	100	50	30	400	300	200	100	50	30
AHI	14.04	13.91	13.63	13.48	13.84	14.12	0.00	0.17	0.25	0.26	0.18	0.02
Height (cm)	5.28	5.23	5.16	5.24	5.46	5.43	0.40	0.41	0.42	0.41	0.29	0.32
Age (years)	9.46	9.22	8.29	8.68	9.10	9.53	0.42	0.51	0.61	0.57	0.50	0.41
Weight (kg)	12.39	12.82	12.18	12.11	12.27	12.59	0.29	0.18	0.32	0.35	0.34	0.24
BMI (kg/m^2^)	3.73	3.70	3.66	3.68	3.72	3.77	0.20	0.18	0.27	0.27	0.21	0.14
CP (cm)	2.38	2.42	2.32	2.34	2.42	2.44	0.31	0.26	0.42	0.40	0.31	0.26

*AHI* apnea–hypopnea index, *BMI* body mass index, *CP* cervical perimeter

The correlation coefficients (ρ) are significant beyond the 0.01 level of confidence

As pointed out before, for supervectors (Table 5), only a linear kernel was evaluated because the very large supervector dimension (>1000) makes not advisable mapping this data into a higher dimensional space.

Tables 6 and 7 show that for i-vectors, estimation results using linear and RBF kernels are very similar. These tables also show that both i-vectors and supervectors reach similar results for almost all clinical variables.

### AHI classification

Table 8 shows classification results in terms of sensitivity, specificity and area under the ROC curve when classifying our population as OSA subjects or healthy individuals based on the estimated AHI values. That is, first supervectors or i-vectors are used to estimate the AHI using SVR, and then subjects are classified as OSA individuals when their estimated AHI is above ten, otherwise they are classified as healthy. The results in Table 8 using i-vectors were obtained for i-vector dimensionality of 100 as this provided the best AHI estimation results (see Table 6).

**Table 8:** OSA Classification using estimated AHI values

Feature	Accuracy (%)	Sensitivity (%)	Specificity (%)	ROC AUC
Supervectors	68	89	18	0.58
I-vectors (dim 100)	71	92	20	0.64

We are aware that better results could be obtained using supervectors or i-vectors as inputs to a classification algorithm such as SVM, however results in Table 8 were only obtained looking for some figures that will be used in the section “Discussion” to compare our results with those from previous research (Table 9).

**Table 9:** Test characteristics of previous research using speech analysis and machine learning for AHI classification and regression

Study	Population characteristics	Classification			Regression
		Correct classification rate (%)	Sensitivity (%)	Specificity (%)	Correlation coefficient
GMMs [10]	80 male subjects (AHI <10: 40 men, AHI >30: 40 men)	81	77.5	85	_
HMMs [11]	80 male subjects (AHI <10: 40 men, AHI >30: 40 men)	85	_	_	_
Several feature selection and classification schemes [13]	248 subjects (AHI ≤5: 48 male, 79 women; AHI ≥30: 101 male, 20 women)	82.85	81.49	84.69	_
Feature selection and GMMs [9]	93 subjects (AHI ≤5: 14 female; AHI >5: 19 female) (AHI ≤10: 12 male; AHI >10: 48 male)	_	86 83	84 79	_
Feature selection and GMMs [41]	103 male subjects (AHI ≤10: 25 male; AHI >10: 78 male)	80	80.65	80	_
Feature selection, supervectors and SVR [14]	131 males	_	_	_	0.67^a^
I-vectors/supervectors and SVR this study	426 males (AHI <10: 125 male; AHI ≥10: 301 male)	71.06	92.92	20.6	0.30

^a^Results using speech features plus age and BMI

## Discussion

Overall, results in Tables 5–7 indicate a poor performance when estimating AHI from acoustic speech information; the best results are obtained using SVR after i-vectors acoustic representation with dimensionality 100 (ρ = 0.30). Better performance is obtained when predicting the other clinical variables: best results were for i-vectors and SVR linear kernel (see Table 6) with correlation coefficient ρ = 0.63 for age followed by CP (ρ = 0.49), height (ρ = 0.45), weight (ρ = 0.39) and BMI (ρ = 0.33).

Nevertheless, the most interesting discussion arises when comparing these results with those reported in previous research.

As stated before our results when estimating age and height are comparable to those previously published in [16] and [17]. Previous research has also demonstrated moderate results (similar to ours) when estimating speakers’ weight and CP from speech (for example, see [33] and [34]). The less success when estimating BMI has also been reported in [35]. Only more positive results have been recently presented in [20], although they have been questioned for possible overfitting by their authors, as they used machine learning after feature selection over a large set of acoustic features.

However, our AHI estimation results contrast markedly with those reported in previous research connecting speech and OSA. Therefore we decided to address a critical review of previous studies (including ours) that led us to identify possible machine learning issues similar to those reported in [19].

A first discrepancy, though not related with machine learning issues, was addressed in our research [36] were we found notable differences with the seminal work by Robb et al. [8]. In [8] statistical significant differences between OSA and non-OSA speakers were found for several formants frequencies and bandwidths extracted from sustained vowels, while our study in [36] only revealed very weak correlations with two formant bandwidths. In this case, the discrepancy can be mainly attributed to the small and biased sample in Robb’s exploratory analysis (10 OSA and 10 no-OSA subjects, including extreme AHI differences between individuals); while in our study [36] we explored a larger sample of 241 male subjects representing a wide range of AHI values.

Table 9 summarizes the most relevant existing research proposals using automatic speech analysis and machine learning for OSA assessment.

We start by reviewing our own previous positive results presented in [10–12]. In [10] and [11] speech samples from control (AHI <10) and OSA (AHI >30) individuals were used to train a binary machine learning classifier for severe OSA detection. Healthy and OSA speakers were thus classified using two models: one trained to represent OSA voices and the other to model healthy voices. Two different approaches were researched: (1) a text-independent approach using two GMMs [10], and (2) through two text-dependent Hidden Markov Models (HMMs) [11]. Correct classification rates were 80 and 85 %, for GMMs and HMMs respectively. These promising results contrast with both the weak correlation between speech and AHI and the low OSA classification performance we have found in this study. Consequently, we repeated experiments in [10] and [11] on the same database used in this paper, and found that performance has now been significantly degraded only achieving correct classification rates of 63 % for GMMs and 67 % for HMMs. This important reduction in performance can again be attributed to the very limited database (40 controls and 40 OSA speakers with AHI >30) used in [10] and [11], while now we have 125 controls (AHI <10) and 118 OSA subjects (AHI >30). As pointed out in [19] the size of training and evaluation sets are important factors to gain a reasonable understanding of the performance of any classifier. Furthermore, another relevant factor that can explain this degradation in performance is that those 40 controls in [10] and [11] were asymptomatic individuals, selected trying to have both control and OSA populations as matched as possible in terms of age and BMI. While in our new database all individuals (i.e., controls and OSA) are suspected to suffer from OSA as they have been referred to a sleep disorders unit (as indicated before control population was defined by AHI <10), so, for example, most of them are heavy snorers. A third possible cause to explain previous over-optimistic results can be traced considering possible indirect influences of speech and AHI mediated through other clinical variables (see correlation coefficients between AHI and other clinical variables in Table 10). More specifically, as it was discussed in [9] speech acoustic features can be less correlated with AHI than with some clinical variables as age or BMI that are good predictors of AHI [37]. Therefore, a population of controls and OSA speakers with significant differences in these confounding variables can be prone to false discovery of discrimination results due to the underlying differences in these confounders and not in AHI. This fact was reported in our research [12] were OSA detection results using 16 speech features (many of them similar to those traditionally used in detecting voice pathologies, such as HNR, Jitter, Shimmer,…) were degraded when tested on a database designed to avoid statistically significant differences in age and BMI.

**Table 10:** Spearman’s correlation between clinical variables

Feature	AHI	Weight	Height	BMI	Age	CP
AHI	1	0.41^a^	−0.007	0.44^a^	0.16^a^	0.40^a^
Weight	0.41^a^	1	0.40^a^	0.89^a^	−0.11^a^	0.71^a^
Height	−0.007	0.40^a^	1	−0.02	−0.35^a^	0.13^a^
BMI	0.44^a^	0.89^a^	−0.02	1	0.04	0.72^a^
Age	0.16^a^	−0.11^a^	−0.35^a^	0.04	1	0.16^a^
CP	0.40^a^	0.71^a^	0.13^a^	0.72^a^	0.16^a^	1

^a^The correlation coefficients (ρ) are significant beyond the 0.01 level of confidence

Same critical demands to explore and report on significant differences in confounding speaker’s features such as age, height, BMI, etc., must be extended to any other factor that could affect speech such as speakers’ dialect, gender, mood state, and so forth. In fact we believe this is an issue that can explain the good discrimination results when detecting severe OSA reported in [13]. The study by Solan-Casals et al. [13] analyzes both sustained and connected speech and recordings from two distinct positions, upright or seated and supine or stretched. The reason for recording two distinct uttering positions, which was also preliminary explored in [15], is that due to anatomical and functional abnormalities in OSA individuals different body positions can affect differently their vocal tract, therefore presenting more discriminative acoustic features. Solan-Casals et al. evaluate several feature selection, feature combination (i.e., PCA) and classification schemes (Bayesian Classifiers, KNN, Support Vector Machines, Neural Networks, Adaboost). Best results are achieved when using a genetic algorithm for feature selection. An interesting result in [13] is that positive discrimination results, i.e., Correct Classification Rate, Sensitivity and Specificity, all above 80 %, were only obtained when classifying between extreme cases: severe OSA (AHI ≥30) and controls (AHI ≤5). While a notable reduction in performance was obtained when trying to classify “in-between cases”, i.e., cases with AHI between 5 and 30. Solan-Casals et al. conclude that “for intermediate cases where upper-airway closure may not be so pronounced (thus voice not much affected), we cannot rely on voice alone for making a good discrimination between OSA and non-OSA.”

At first glance, this conclusion of [13] could be linked to our weak estimation and classification results for the broad range of AHI values using acoustic speech information. However, there are two critical issues that can be identified in this study. First, feature selection is applied over a high number of features (253) compared to the number of cases (248). Though authors report the use of cross-validation for the development and evaluation of different classification algorithms there is no clear indication on what data was used for feature selection. At this point, it is worth noting that i-vectors subspace projection in our study was trained using a development database completely different from the one used for training and testing our SVR regression model. Without this precaution, as discussed in several studies [19, 38], feature selection can lead to over-fitted results based on a set of “ad-hoc” selected features. A second highly relevant issue in [13] is that when evaluating the classification performance between extreme cases (see Table 7 in [13]), OSA and control groups contain very different percentages of male and female speakers: 48 men/79 women in control vs. 101 men/20 women in OSA. This notable imbalance between female and male percentages in control and OSA groups is clearly due to the significantly lower prevalence of OSA in women compared to men [39]. Consequently, considering the important acoustic differences between female and male voices [40], this makes gender a strong confounding factor that could also explain the good classification results. To illustrate these issues, we have studied the best discriminative feature reported in [13] which is the mean value of the Harmonics to Noise Ratio (HNR) measured for sustained vowel/a/recorded in seated position (MEAN_HNR_VA_A in [13]). A small *p* value, p < 0.0001, was reported in [13] using a Wilcoxon two-sampled test of difference in medians for MEAN_HNR_VA_A values in control and OSA groups. As our database also contains speech records of sustained/a/recorded in seated position for both 426 male individuals and 171 female speakers, we have made Wilcoxon two-sampled tests for MEAN_HNR_VA_A values contrasting: a) a group of male speakers vs a group of female speakers, and b) a group of extreme OSA male speakers (AHI ≥30) with another of male controls (AHI ≤5). Results presented in Table 11, clearly reveal that while significant differences (p < 0.0001) appear contrasting female and male voices (which has already been reported in other studies such as [40]), no significant differences are found between extreme OSA groups including only male speakers (p = 0.06). This is therefore an illustrative example on how gender can act as a strong confounding factor.

**Table 11:** Wilcoxon two-sampled test for MEAN_HNR_VA_A contrasting gender and group of extreme OSA male speakers

	Mean_HNR_VA_A (Gender)			Mean_HNR_VA_A (extreme OSA male speakers)		
	Female	Male	p value	Male (AHI ≤5)	Male (AHI ≥30)	p value
Median	19.43	17.07	<0.0001	17.46	16.38	0.06
SD	3.98	4.23		3.89	4.32	
# Samples	171	426		69	129	

The connection between OSA and speech analysis has also been studied for Hebrew language, mainly in [9] and [14]. Following the same approach previously described for [10], the work in [9] uses two GMMs to classify between OSA and non-OSA speakers. However, differently from [10] acoustic feature selection is made before GMM modelling. The experimental protocol presented by Goldshtein et al. in [9] properly separates female and male speakers. Different AHI thresholds are used to define OSA and non-OSA groups: an AHI threshold of 5 is used for women and 10 for men. Reported results achieved specificity of 83 % and sensitivity of 79 % for OSA detection in males and 86 and 84 % for females (see Table 9). A major limitation in this study is again the small number of cases under study: a total number of 60 male speakers (12 controls/48 OSA) and 33 female subjects (14 controls/19 OSA). Besides the low reliability with such small samples, again a critical issue, both in [9] and [14], is the use of feature selection techniques from a large number of acoustic parameters (sometimes on the order of hundreds) when only very limited training data is available. The same research group reported in [41] a decrease in performance using the same techniques as in [9] but over a different database with 103 males. According to Kriboy et al. in [41], this mismatch could be explained by the use of a different database with more subjects and with a different balance in terms of possible confounding factors BMI, age, etc.

Also particularly relevant can be to analyze the good results estimating AHI reported by Kriboy et al. in [14] because they used a prediction scheme very close to the one we have presented in this paper: GMM supervectors are used in combination with SVR to estimate AHI. Nevertheless, differently from our study, again feature selection is firstly used to select the most five discriminative features from a set of 71 acoustic features, and then GMM mean supervectors are trained for that small number of features. Although the experimental protocol in [14] separates training and validation data to avoid over-fitting, the set of selected features was composed by five high-order cepstral and LPC coefficients (a15, ΔΔc9, a17, ΔΔc12, c16) which are difficult to interpret or justify. Both cepstral and LPC coefficients are commonly used to represent the acoustic spectral information in speech signals, but higher order coefficients are generally less informative and noisy. Another notable limitation to validate results in [14] is that SVR regression is applied after adding two clinical variables, age and BMI, to the speech supervector generated from the five selected features. These two clinical variables are well known predictors of AHI [37]. So it should had been advisable first to report AHI estimation results only using supervectors representing speech acoustic features, then to present results only using age and BMI, and finally give results extending supervectors with age and BMI.

Trying to contribute to review these results we have applied the same estimation procedure described in [14] to our database. First row in Table 12 shows prediction results for AHI using only speech supervectors including the same set of five selected features in [14]. Second row presents estimation performance when using only BMI and age. Third row includes the results using the supervector of acoustic features extended with BMI and age.

**Table 12:** Speakers’ AHI estimation using supervector generated by five high-order cepstral and LPC coefficients [14]

Set of clinical variables	MAE	Correlation coefficient (ρ)	*p* value
a15, ΔΔc9, a17, ΔΔc12, c16	14.33	0.12	0.008
AGE + BMI	12.96	0.38	<0.00001
(a15, ΔΔc9, a17, ΔΔc12, c16) + AGE + BMI	12.24	0.46	<0.00001

p values are given for correlation coefficient (ρ)

As it can be seen in Table 12, estimation results are mainly driven by the presence of BMI and age, and very poor correlation (ρ = 0.12) is obtained when only the set of 5 selected speech features is used. Therefore, it is reasonable to conclude that the well-known correlation between AHI and BMI and age [37, 42] together with possible over-fitting from feature selection on a high number of features compared to the number of cases can cause the optimistic results presented in [14].

We acknowledge several limitations in our work that should be addressed in future research. Results presented in this paper are limited to speech from Spanish speakers, so comparisons with other languages will require a more careful analysis of language-dependent acoustic traits in OSA voices. Another limitation in our study is that it has only considered male speakers. As our database now includes an important number of female speakers the extension of this study on female voices could be especially interesting as apnea disease is still not well researched in women. Considering also some recent studies as [43], we should also acknowledge the limitation of i-vectors to represent relevant segmental (non-cepstral) and supra-segmental speaker information. Therefore, subspace projection techniques could also be explored over other speech acoustic features previously related to OSA as: nasality [9, 10], voice turbulence [13, 44] or specific co-articulation trajectories. Finally, a comparative analysis of results for both different recording positions (as proposed in [15]) should be addressed.

## Conclusions

This study can represent an important and useful example to illustrate the potential pitfalls in the development of machine learning techniques for diagnostic applications. The contradictory results using state-of-the-art speech processing and machine learning for OSA assessment over, to the best of our knowledge, the largest database used in this kind of studies, led us to address a critical review of previous studies reporting positive results in connecting OSA and speech. As it is being identified in different fields by the biomedical research community, several limitations in the development of machine learning techniques were observed and, when possible, experimentally studied. In line with other similar studies on these pitfalls [19, 38] main detected deficiencies are: the impact of a limited size of training and evaluation datasets in performance evaluation, the likelihood of false discovery or spurious associations due to the presence of confounding variables, and the risk for overfitting when feature selection techniques are applied over large numbers of variables when only limited training data is available.

In conclusion, we believe that our study and results could be useful both to sensitize the biomedical engineering research community to the potential pitfalls when using machine learning for medical diagnosis, and to guide further research on the connection between speech and OSA. In this later aspect, we believe there is an open way for future research looking for new insights in this connection using different acoustic features, languages, speaking styles, or recording positions. However, besides properly addressing the methodological issues when using machine learning, any new advance should carefully explore and report on any possible indirect influence of speech and AHI mediated through other clinical variables or any other factor that could affect speech such as speakers’ dialect, gender or mood state.

## Abbreviations

AHI: apnea–hypopnea index
BMI: body-mass index
CP: cervical perimeter
DCT: discrete cosine transform
DFT: discrete Fourier transform
GMM: Gaussian mixture model
HMM: hidden Markov model
HNR: harmonics to noise ratio
KNN: K-nearest neighbor
LOO: leave-one-out
MAE: mean absolute error
MAP: maximum a posteriori
MFCCs: Mel-frequency cepstral coefficients
ΔMFCCs: velocity Mel-frequency cepstral coefficients
OSA: obstructive sleep apnea
PCA: principal components analysis
PSG: polysomnography
ROC: receiver operating characteristic
RBF: radial basis function
SVR: support vector regression
SVM: support vector machine
UA: upper airway
